# Epilepsy Diagnosis from EEG Signals Using Continuous Wavelet Transform-Based Depthwise Convolutional Neural Network Model

**DOI:** 10.3390/diagnostics15010084

**Published:** 2025-01-02

**Authors:** Fırat Dişli, Mehmet Gedikpınar, Hüseyin Fırat, Abdulkadir Şengür, Hanifi Güldemir, Deepika Koundal

**Affiliations:** 1 Department of Electrical and Electronic Engineering, Faculty of Technology, Firat University, 23000 Elazig, Turkey; dislifirat@gmail.com (F.D.); mgedikpinar@firat.edu.tr (M.G.); ksengur@firat.edu.tr (A.Ş.); hguldemir@firat.edu.tr (H.G.); 2 Department of Computer Engineering, Faculty of Engineering, Dicle University, 21000 Diyarbakir, Turkey; 3 A.I. Virtanen Institute for Molecular Sciences, University of Eastern Finland, 70211 Kuopio, Finland; dkoundal@uef.fi

**Keywords:** epilepsy, depthwise convolution, image concatenate, continuous wavelet transform

## Abstract

**Background/Objectives:** Epilepsy is a prevalent neurological disorder characterized by seizures that significantly impact individuals and their social environments. Given the unpredictable nature of epileptic seizures, developing automated epilepsy diagnosis systems is increasingly important. Epilepsy diagnosis traditionally relies on analyzing EEG signals, with recent deep learning methods gaining prominence due to their ability to bypass manual feature extraction. **Methods:** This study proposes a continuous wavelet transform-based depthwise convolutional neural network (DCNN) for epilepsy diagnosis. The 35-channel EEG signals were transformed into 35-channel images using continuous wavelet transform. These images were then concatenated horizontally and vertically into a single image (seven rows by five columns) using Python’s PIL library, which served as input for training the DCNN model. **Results:** The proposed model achieved impressive performance metrics on unseen test data: 95.99% accuracy, 94.27% sensitivity, 97.29% specificity, and 96.34% precision. Comparative analyses with previous studies and state-of-the-art models demonstrated the superior performance of the DCNN model and image concatenation technique. **Conclusions:** Unlike earlier works, this approach did not employ additional classifiers or feature selection algorithms. The developed model and image concatenation method offer a novel methodology for epilepsy diagnosis that can be extended to different datasets, potentially providing a valuable tool to support neurologists globally.

## 1. Introduction

Epilepsy is a neurological disorder that affects millions of people worldwide. It occurs in the form of seizures with fainting and muscle spasms accompanied by electrical activity disorder in the brain [1]. During an epileptic seizure, the individual loses physical and mental control. Therefore, the quality of life as well as economic and social activities of the individual with epilepsy are negatively affected [2]. Although some of the reasons that trigger epileptic seizures are known, the exact time of attack is not known. Considering these situations, studies on epilepsy diagnosis gain importance and attract the interest of researchers [3].

Since the exact timing of an epileptic seizure is unknown, automatic diagnosis of epilepsy seizures using advanced methods is becoming more important than manual diagnosis [4]. Since the electrical activity of the brain can be monitored by EEG signals, the analysis of EEG signals is primarily used for automatic diagnosis of epileptic seizures. In addition, structural and functional neuroimaging techniques, such as magnetic resonance imaging (MRI), computed tomography (CT), positron emission tomography (PET), single photon emission computed tomography (SPECT) scans, are also frequently used [5,6]. While the relatively inexpensive acquisition of EEG signals compared to other methods provides an advantage, the importance of sensitivity in the analysis of the signal and the scarcity of personnel to analyze it constitute disadvantages. However, with automated advanced methods, disadvantages can be overcome and diagnosis time can be accelerated [7]. Nowadays, the use of machine learning and deep learning algorithms has become widespread in studies on automatic advanced methods [8].

In this study, a continuous wavelet transform-based depthwise convolutional neural network (CWT-based DCNN) method was proposed for epilepsy diagnosis from EEG signals, using continuous wavelet transform (CWT) and a depthwise convolutional neural network (DCNN) model. The experimental studies used a publicly available 35-channel EEG dataset for epilepsy. In addition, unlike previous studies, image fusion was used along both the horizontal axis and the vertical axis.The contributions of the proposed CWT-based DCNN method are as follows:

- The study integrates continuous wavelet transform (CWT) with depthwise convolutional neural networks (DCNN), providing an effective framework for analyzing EEG signals in epilepsy diagnosis. The use of CWT enables time-frequency representation of EEG signals, capturing critical temporal and spectral features, which are essential for accurate diagnosis. The DCNN leverages depthwise separable convolutions, reducing computational complexity while maintaining high performance.
- A unique image fusion approach is proposed, combining features along both the horizontal and vertical axes. This fusion enhances feature richness and improves the robustness of the input representations for the DCNN model. According to the experimental results, the proposed method has achieved higher success compared to both state-of-the-art models and previous studies using the same dataset.
- The lightweight nature of the DCNN architecture and its compatibility with EEG data make the method suitable for deployment in clinical settings.

The remainder of the study is organized as follows. In Section 2, a general summary of studies related to machine learning and deep learning in the literature is presented. Section 3 includes the proposed method, the theoretical background of the proposed method, and the dataset used in the study. In Section 4, the experimental studies and the results of these experimental studies are explained in detail. In Section 5, comparisons are made with different studies in the literature that use the same dataset. Finally, in Section 6, a general summary of the study is provided.

## 2. Related Works

Machine learning and deep learning-based approaches have come to the forefront for the automated diagnosis of epilepsy using EEG signals. Deep learning methods provide high accuracy rates due to automatic feature extraction in large datasets. On the other hand, machine learning methods provide fast results in small datasets. In addition, machine learning methods are used in feature extraction or labelling tasks. There are hybrid methods that combine the advantages of both methods in the literature [9].

### 2.1. EEG Classification Using Machine Learning

Machine learning approaches are effective methods for feature extraction from EEG signals and the classification of extracted features. Support vector machine, decision tree, K-nearest neighbours (kNN) algorithms are machine learning algorithms widely used by researchers in the literature. For example, Ra et al. [10] studied EEG channel selection to improve the accuracy of epileptic seizure prediction and reduce the computational cost. They integrated permutation entropy, K-nearest neighbors and genetic algorithm methods in the channel selection phase and then classified them with SVM. In their study, they obtained an improvement in the sensitivity (23.57%) and specificity (5.56%) of the predictions made using channel selection compared to predictions using all channels. In a similar study, Zhou and Wang [11] used spatio-temporal adaptive diffusion models (STADMs) to convert a low-resolution 64 or less channel EEG signal into a 256-channel high-resolution EEG signal to overcome limitations in epilepsy diagnosis. They emphasized that their proposed method improves the performance and reduces the cost of existing methods in epilepsy diagnosis tasks. In a recent study, Tasci et al. [12] implemented epilepsy detection using a novel hypercube-based information fusion model. As a method, feature vectors are extracted from EEG signals using multilevel discrete wavelet transform (MDWT) and a hypercube-based feature extractor. Then, these feature vectors were fed into a (kNN) classifier and tested with cross-validation. They obtained 87.78% classification accuracy with their proposed method. In another study, Kumar et al. [13] computed a histogram of local variance (HLV) and symmetrically weighted-local binary pattern (SLBP) features from EEG signals and classified them using AdaBoost. They tested their method on two different datasets and obtained 92.85% and 99.36% classification accuracy.

In some studies, the features of machine learning methods have been improved by using different techniques. For example, Al-Hadeethi et al. [14] used the adaptive boosting least square-support vector machine (AdaBoost LS-SVM) method to classify time series signals for epileptic seizure diagnosis. In their study, they used a combination of the boosting method to improve the performance of weak classifiers and evaluated the performance of their proposed method with metrics such as accuracy, precision, recall and F1 score. In their results, they showed that the LS-SVM approach supported by adaptive boosting achieves higher accuracy rates compared to traditional methods and provides an effective solution for epileptic seizure detection. In another study, Xu et al. [15] proposed a method using the nonlinear features of the EEG signals and a gradient boosting tree classifier (GBDT) for the early detection of epileptic seizures. Nonlinear features of the EEG signal, approximate entropy, sample entropy, permutation entropy, spectral entropy and wavelet entropy, were extracted to be used in the classification process. They obtained approximately 92% accuracy with the GBDT classifier they proposed, extracting features from EEG signals of different durations.

There are many studies that combined more than one machine learning method in order to reduce the result errors [16]. For example, Dastgoshadeh and Rabiei [17] used least-squares support-vector machine (LS-SVM), K nearest neighbours (KNN) and naive Bayes methods in their study. Firstly, they extracted useful information from the input signal using discrete wavelet transform (DWT). Then they ranked the extracted features with ANOVA test. In the last stage, they used a forward sequential feature selection technique (FSFS) to classify the features they selected with an ensenble machine learning structure. The results of their studies have shown that the proposed ensemble model can be effective in covering errors compared to the learning models used separately and can increase the detection accuracy by at least 1.5%.

### 2.2. EEG Classification Using Deep Learning

Deep learning models do not require additional processing for feature extraction and pre-trained models can be used for other problems by transfer learning. Due to these advantages, the use of deep learning methods in the classification of EEG signals has come to the forefront in recent years. For example, Lih et al. [18] trained the Epilepsy-Net neural network, a deep learning-based transformer model. They transformed the data segments obtained from EEG signals into new matrices with the Pearson correlation coefficient (PCC) and performed classification using 10-fold validation. They reported that their proposed model requires less computation than existing models and performs at about 85% classification accuracy. In another study, Raghu et al. [19] tried to solve the multi-class seizure type classification problem using transfer learning and achieved around 88% accuracy. In addition to transformer models, CNN models with high performance in image classification problems are preferred by researchers. Palanisamy et al. [20] proposed an effective system to analyze and recognize encrypted EEG information using Arnold transform algorithms, chaotic mapping and CNNs. First, they encrypted the time series EEG signal with chaotic algorithms and converted it into a 2D spectrogram image. Then, the encrypted data was processed by CNNs combined with transfer learning (TL) frameworks. They tried their classifiers with different optimized parameters and achieved a final F1 score of 98.80%.

In order to increase the effectiveness of the models, the combined structures of more than one deep learning structure are used. Islam et al. [21] studied a dynamic model for epileptic seizure detection using a deep learning model (Epileptic-Net). Their proposed method consists of dense convolutional blocks (DCB), residual blocks (RB), feature attention modules (FAM) and the hypercolumn technique (HT). Firstly, DCB was used to extract discriminative features from EEG samples. Using Fa10-fold validation, they evaluated their model on the Bonn University EEG dataset divided into five different classes. They achieved around 99% classification success for three-class, four-class and five-class problems. In a similar study, Najafi et al. [22] proposed an artificial neural network model combined with RNN and long short-term memory LTSM for epilepsy diagnosis. They used discrete wavelet transform (DWT) and longitudinal bipolar assembly (LB) techniques for feature extraction. When the features extracted from normal and epileptic samples were classified with their proposed model, they achieved approximately 96% accuracy.

For the training of deep learning models, the datasets need to be large enough. For this reason, some researchers are working on data augmentation methods. Zhang et al. [23] focus on the generalization problem in deep learning approaches. First, they proposed spatiotemporal augmentation (STEA) as a data augmentation method to greatly improve generalization. Then, they used the patient-adversarial neural network (PANN) for classification. They tested their proposed methods on both public and clinical EEG datasets with different window lengths and partitions of training and test sets. As a result, it achieves up to 95% precision on CHB-MIT in 5-s window length segments, and reaches about 85% AUC and precision even on a clinical dataset. Deep learning models trained on large datasets are widely used for different problems. Silveria and Correa [24] proposed a transfer learning-based approach. They created images from the epilepsy dataset by graphically plotting its data points to train their model. As a result of their study, they achieved 100% accuracy and drew attention to the potential of transfer learning.

In addition to the diagnosis of epileptic seizures, machine learning and deep learning methods are used to assist the diagnosis process in many medical fields such as autism, schizophrenia, liver tumors, cancer, heart and eye diseases [25,26,27,28,29,30,31,32,33].

## 3. Proposed Method

**Figure 1:**
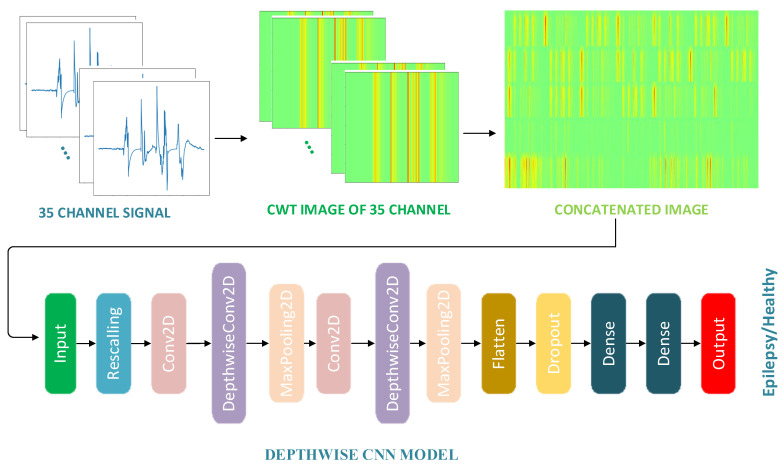
Proposed method.

Considering the recent studies, it is predicted that epilepsy diagnosis can be made by processing EEG signals with continuous wavelet transform (CWT) and classifying them with depthwise convolutional neural network (CWT-based DCNN) model. In this study, a new model is proposed for classification on a publicly available epilepsy dataset containing 35 channels of EEG signals. This model contains three main components. The first component is CWT to extract the image features of the 35-channel signals. The second component is the concatenation process which is used in order to avoid any feature loss in the obtained images. The third component is the depthwise-based CNN architecture used for epilepsy classification. Figure 1 shows a visualization of the proposed method. The process steps of the proposed method for epilepsy diagnosis are as follows.

1. Preparation of the dataset,
2. Image generation from 35-channel EEG signals using CWT,
3. Concatenating the images of 35 channels into a single image,
4. Development of the DCNN model,
5. Classification of the images with the developed DCNN model.

In this study, a CNN model with depthwise convolution layers is proposed for epilepsy diagnosis. Information about the proposed model is given in Table 1. The rescaling layer, which converts 500 × 700 × 3 images to 150 × 210 × 3, is used as the first layer. Conv2d, depthwise and maxpooling layers are used in the intermediate layers, two of each. Relu was chosen as the activation function in the intermediate layers and the dropout rate used was 0.6. For the classification process, as there are two classes, epilepsy and normal, the activation function of the last layer was chosen as sigmoid.

A detailed description of the different techniques used in the proposed CWT-based depthwise CNN is given as follows.

### 3.1. Continuous Wavelet Transform

Continuous wavelet transform (CWT) is a technique that allows a signal to be analyzed at different time and frequency resolutions. Unlike the Fourier and Hilbert transform, the CWT provides a variable time-frequency resolution to analyze the signal at specific scales. This signal processing technique is particularly successful in analyzing non-stationary and transient signals. The mathematical formula of the CWT can be expressed as follows [34,35,36,37]:

(1) $${\mathrm{CWT}}_{x}(a,b)=\int_{-\infty }^{\infty }x\left(t\right)\frac{1}{\sqrt{\left|a\right|}}\psi \left(\frac{t\;-\;b}{a}\right)\mathrm{dt}$$

**Figure 2:**
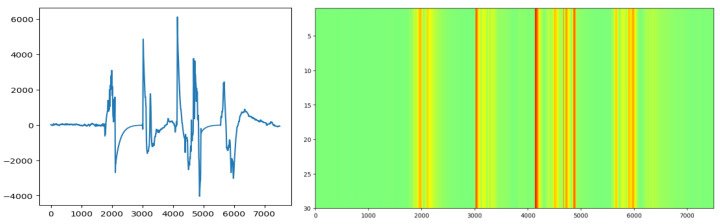
Sample signal and CWT applied form.

In this equation, x(t): analyzed signal, ψ(t): mother wavelet, a: scale parameter (determines the frequency resolution of the signal), b: time shift parameter (determines the time resolution). By varying the scale parameter, different frequency components can be analyzed. Figure 2 shows an example of the CWT transform used in the study applied to a signal. Python “pywt” library was used for the CWT process. The CWT function parameters used are interpolation = “bilinear”, cmap = “jet”, aspect = “auto” and wavelet function “morl”.

### 3.2. Image Concenating

Image concatenation can be defined as the process of merging multiple images to create an image used to provide data for artificial intelligence models. In studies on this subject, it has been observed that combining different visual data belonging to a problem contributes positively to the performance of image classification and object detection models. In practice, the concatenation process can be applied not only by merging pixel values, but also by merging feature maps or layers. Concatenation is usually applied along a single axis, such as end-to-end addition and addition along the vertical axis. The emergence of new relationships between the concatenated images is an advantage of image concatenation over other concatenation techniques [38,39,40,41,42].

**Figure 3:**
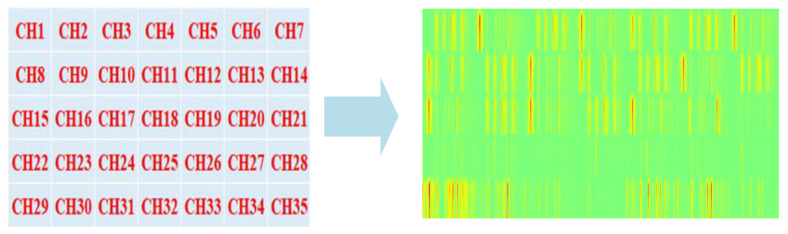
Concenating method.

In this study, images generated from 35-channel EEG signals using CWT were concatenated along the horizontal and vertical axis. First, 100 × 100 resolution images of 35 channels were created. Then, using the Python “PIL” library, 35 100 × 100 resolution images were concatenated to obtain a concatenated image with a resolution of 700 × 500 (width × height). Figure 3 shows the image concatenation method and the concatenated image. The position of a channel’s image in the concatenation process was determined using the channel’s sequence number i. The horizontal position of the channel is computed as pos_x = i%7 and the vertical position as pos_y = math.floor(i/7). Here, % is the mode operation and math.floor() is the round down operation.

### 3.3. Depthwise Separable Convolution

In recent years, there have been significant advances in artificial intelligence systems with advances in computer hardware and software; especially in the field of artificial neural networks, revolutionary changes have occurred in analyzing complex and large datasets. Among these changes, convolutional neural network (CNN)-based models have played an important role in solving image and signal processing problems [43].

**Figure 4:**
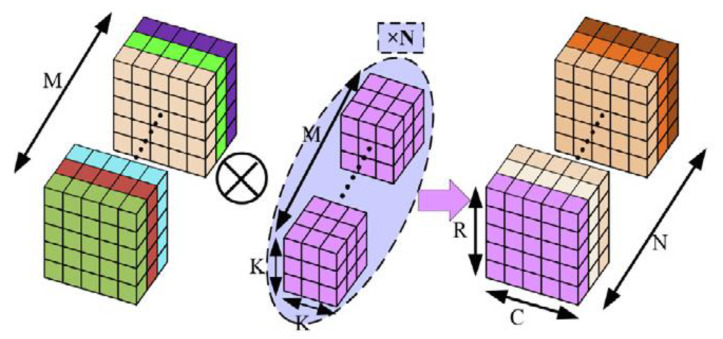
Convolution operation.

CNN models are a class of deep neural networks that are frequently used to process data such as images and signals. They are built as stacks of layers These layers consist of convolutional layers, pooling layers and fully connected layers. Convolutional layers apply a series of filters (kernels) to the input data to extract features such as edges, textures and shapes Pooling layers reduce data size and lighten the computational load by preserving the most important information The ability of CNN architectures to learn hierarchical representations and local features from input data makes them a solution in many domains [44]. Figure 4 shows the principle of the convolution process Where M is the number of channels of the input data, K is the filter size and N is the number of filters. Since each filter is applied to all channels, the depth of the output matrix is equal to the number of filters, N [45].

**Figure 5:**
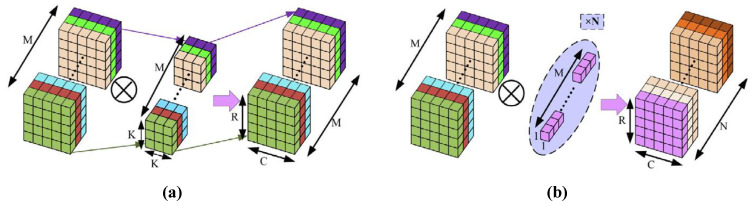
(**a**) Depthwise convolution, (**b**) pointwise convolution.

In conventional 2D convolution, feature maps are created by filtering the input data along both spatial dimensions and all channels. In contrast, in depthwise separable convolution, the convolution process consists of two steps. In the first step, depthwise convolution is applied to each channel independently. In the second step, pointwise convolution (using 1 × 1 filters) is applied, which combines the output channels. Figure 5 shows the depthwise convolution process [31]. The depthwise convolution significantly reduces the number of computations and parameters while preserving performance. Thus, CNN models that normally require high computational capacity can be adapted to mobile devices and embedded systems. For example, CNN models have been optimized for mobile and embedded devices using depthwise convolution in some architectures such as MobileNet, ShuffleNet, Xception [32,43].

### 3.4. Turkish Epilepsy Dataset (Public Dataset)

In this study, a publically available Turkish epilepsy EEG dataset containing 35-channel EEG signals was used [46]. The signals were obtained from 121 individuals, 71 normal and 50 epileptic. Since the signals in the dataset are randomly sampled for 1 s each, the number of samples for each person is different. For example, a person with epilepsy has 48 samples while another person has 34 samples. Similarly, a normal person has 61 samples, while another person has 205 samples. In total, the dataset contains 10356 35-channel signal samples [12].

## 4. Experimental Results

### 4.1. Experimental Setup

Experimental studies were carried out in Kaggle Jupyter Notebook environment with Python programming language. The CWT-based DCNN model was developed and trained using 100 epochs with a batch size of 128, binary cross entropy loss function and Adam optimization algorithm with a learning rate of 0.0002. The dataset used was divided into two parts as training and test data, 75% of the data was used for training and 25% of the data was used for both testing and validation.

### 4.2. Evaluation Criteria

The accuracy, sensitivity, specificity, precision and f1-score values were used to assess the performance of the CWT-based DCNN model. These performance metrics provide an objective and quantitative measure of the predictive efficiency of the model, which is necessary for performance evaluation and identification of areas for improvement. The equations of these metrics are given in Equations (2)–(6).

(2) $$\mathrm{Accuracy}=\frac{\mathrm{TP}+\mathrm{TN}}{\mathrm{TP}+\mathrm{TN}+\mathrm{FP}+\mathrm{FN}}$$

(3) $$\mathrm{Sensitivity}=\frac{\mathrm{TP}}{\mathrm{TP}+\mathrm{FN}}$$

(4) $$\mathrm{Specificity}=\frac{\mathrm{TN}}{\mathrm{TN}+\mathrm{FP}}$$

(5) $$\mathrm{Precision}=\frac{\mathrm{TP}}{\mathrm{TP}+\mathrm{FP}}$$

(6) $$\text{F1-score }=\frac{2\;\times \;\mathrm{Precision}\;\times \;\mathrm{Sensitivity}}{\mathrm{Precision}+\mathrm{Sensitivity}}$$

Equations (2)–(5) from the confusion matrix is derived true positives (TP), false positives (FP), false negatives (FN) and true negatives (TN). These represent the following: TP is the number of correctly predicted as epileptic, TN is the number of correctly predicted as normal, FP is the number of incorrectly predicted as normal, FN is the number of incorrectly predicted as epileptic [47].

### 4.3. Results

**Figure 6:**
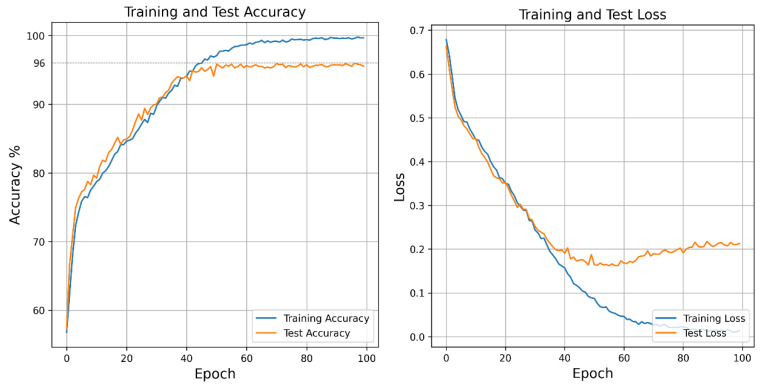
Training–test process of the CWT-based DCNN model.

The proposed CWT-based DCNN model was trained using different input sizes, different learning rates and different optimizers. The highest performance was achieved when the input size was 150 × 210 (height × width), the optimizer was Adam and the learning rate was 0.0002. The plot of this training process is shown in Figure 6. As seen in this figure, it can be said that the model has learned the data. In the 20th epoch, the training accuracy was over 80%, in the 40th epoch over 90% accuracy was achieved and in the 60th epoch over 95% accuracy was observed. From the 40th epoch, overfit was observed and a validation accuracy of 95.99% was reached at the end.

**Figure 7:**
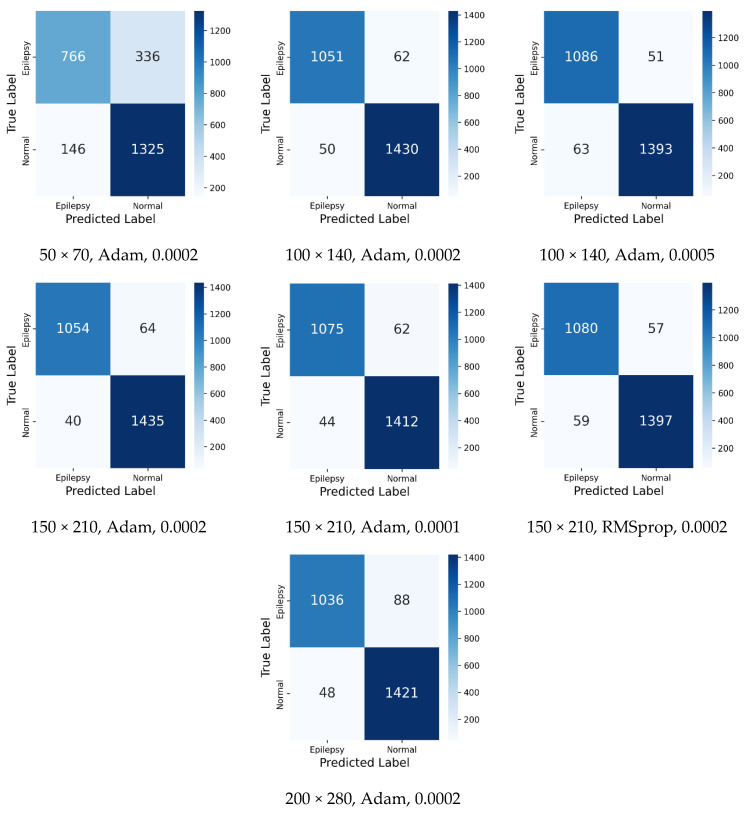
Confusion matrix of the proposed CWT-based DCNN model with different parameters (input size, optimizer, learning rate).

Figure 7 shows the confusion matrix of the test results of training the model with different parameters. The parameters and evaluation metrics of these experiments are given in Table 2. Also, the order of the confusion matrices and the order of the trials in this table are the same. When the confusion matrices are examined, 766 correct and 336 incorrect predictions of the epilepsy class were made as a result of training the model with the lowest input size 50 × 70, with a learning rate of 0.0002 parameters. On the other hand, with the largest input size 200 × 280, a learning rate of 0.0002 parameters, in the epilepsy class, 1036 correct and 88 incorrect predictions were made. The highest classification value of the epilepsy class was obtained with 1086 correct and 51 incorrect predictions in the training with 100 × 140 input size, a learning rate of 0.0005 parameters. The 1051 correct and 62 incorrect predictions were obtained for the epilepsy class in the training with 100 × 140 input size and learning rate 0.0002 parameter.

In the training with 150 × 210 input size, Adam of 0.0002, Adam of 0.0001, and RMSprop of 0.0002 were used, respectively, as optimizer and learning rate. In these experiments, 1054, 1075, 1080 correct predictions and 64, 62, 57 incorrect predictions were obtained for the epilepsy class, respectively.

For the results of the classification of the class labelled as normal, when the confusion matrix is examined, the highest classification was obtained with 1435 correct and 40 incorrect predictions with a 150 × 210 input size and 0.0002 learning rate parameters. In the other two experiments for the same input size, 1412 and 1397 correct predictions and 44 and 59 incorrect predictions were obtained. The lowest normal classification value was obtained with 1325 correct predictions and 146 incorrect predictions with a 50 × 70 input size. In the experiments performed with learning rate parameters of 0.0002 and 0.0005 for the input size of 100 × 140, 1430 and 1393 correct predictions and 50 and 63 incorrect predictions were obtained. In the experiment with the largest input size used in the study, 200 × 280, 1421 correct and 48 incorrect predictions were obtained for the labeled class normal.

When the confusion matrices are examined, it can be seen that the model is better at classifying the data labelled as normal. This result can be reinforced by the fact that the specificity value is above 90% in all trials. For this reason, the sensitivity value increases in importance as well as the accuracy value in the performance evaluation of the method proposed in this study. Although the highest accuracy value of 95.99% was obtained with 150 × 210 input size, learning rate of 0.0002 parameters, the highest sensitivity value of 95.51% was obtained with 100 × 140 input size and a learning rate of 0.0005 parameters. When all the results are analyzed, it is seen that high classification accuracy, sensitivity, specificity, precision and f1-score of 95.99%, 95.51%, 97.29%, 96.34% and 95.30% are obtained for the classification of healthy and epileptic EEG signals.

The results given in Table 2 show that the highest accuracy values obtained according to the input sizes were 81.41% for 50 × 70, 95.68% for 100 × 140, 95.99% for 150 × 210 and 94.75% for 200 × 280. When the results obtained are analyzed, it can be said that specificity, precision and f1-score values are in parallel with accuracy values. For example, the highest specificity values obtained according to the input size are 90.07 for 50 × 70, 96.92 for 100 × 140, 97.29 for 150 × 210 and 96.73 for 200 × 280. The precision values were 83.99% for 50 × 70, 95.46% for 100 × 140, 96.34% for 150 × 210 and 95.57% for 200 × 280, respectively. In addition, the highest f1-score values are 76.06% for 50 × 70, 95.01% for 100 × 140, 95.30% for 150 × 210 and 93.84% for 200 × 280. When the results in Table 2 are compared according to the input size, the sensitivity value differs from the other values. The highest sensitivity value is 95.51% for 100 × 140 input size. In addition, the sensitivity values for other input sizes are calculated as 69.51% for 50 × 70, 94.55% for 150 × 210 and 92.17% for 200 × 280.

In addition to Table 2, experiments with the SGD and Adagrad optimizers were performed and no learning was observed. Considering the results with learning, the input size can be shown as the factor that affects success the most. In addition, the discrimination of the results in the experiments was in the classification of the data labelled as epilepsy. Although the highest accuracy was achieved at an input size of 150 × 210, the results of other experiments with high accuracy values are close to this result. The reason for this situation may be the characteristics of the dataset or the limitations of the proposed model. Since the signals in the dataset were generated by randomly sampling long-term signals from participants to be 1 s in length, some signals may have lost their distinctive features for the classification problem used in this study.

The increase in the size of the input data leads to an increase in the number of parameters in the artificial neural network, in other words, the growth of the artificial neural network. The growth of the neural model may increase the overfit effect in the training time. Therefore, the growth of the model may not always increase the test accuracy. The relationship between the increase in the size of the input data and the overfit may explain the fact that the highest accuracy value in the experiments was obtained with 150 × 210 rather than 200 × 280.

## 5. Discussion

As can be seen in the results, a high classification accuracy of 95.99% was achieved when using CWT and image concatenation for epilepsy diagnosis from EEG signals and training with the DCNN model. In other studies using the same dataset, Tasci et al. [12] used the hypercube-based technique to obtain feature vectors, while Lih et al. [18] used the Pearson correlation coefficient and positioning. Instead of these, in this study, the input data was obtained by concatenating images generated from 35-channel EEG signals using scale-based CWT. As a classifier, Tasci et al. [12] used the k-NN classifier and obtained 87.78% accuracy. On the other hand, Lih et al. [18] used the transformer model and achieved 85% accuracy. In contrast to these, in this study, the classification was performed with the DCNN model. In addition to these, classifications were made with some models whose results are shown in Table 3. When the input data and optimizer parameters were selected to be the same as the model proposed in the study and training was performed, maximum accuracy values of 66.71%, 87.42%, 91.13%, 91.66% and 93.75% were achieved with Resnet50, Xception, Vgg16, MobileNet, InceptionV3, respectively. Similarly, f1-score values for the same models were obtained as 70.25%, 85.65%, 89.88%, 90.51%, 92.14%, respectively. It was found that the proposed methods achieved higher success than other studies on the same dataset [48,49,50,51,52].

In the studies in the literature, new layers are added to the neural network models for feature selection or feature fusion, while in this study, no additional layers are used except for the CNN model. By using depthwise layers, a simpler model has been developed compared to the neural networks used in existing studies. In addition, complex methods were not used for data preprocessing and all operations could be performed in a single software environment. In addition, since the signals of all channels are concatenated, there is no loss of any feature. In addition, since new features emerged from the location relations of the concatenated images, high performance was achieved with the developed model. The training–testing accuracy graph and confusion matrix show the good performance of the methods used in the study. The advantages and disadvantages of the study can be given as follows.

Advantages:

1. A high accuracy of 95.99% was achieved using CWT-based DCNN.
2. It is a new method for the dataset used in the study.
3. No feature loss was caused by concenating 35-channel EEG signals.
4. When the obtained results are examined, the proposed method can be helpful for experts in diagnosing epileptic patients.
5. The architecture of the proposed system is simpler than the methods in existing studies.

Limitations:

1. The dataset used in the study contains data from a small number of people.
2. It can be tried on datasets containing signals from epileptic patients at different levels.

In future studies, the methods used in the study can be tried on datasets with different levels of epileptic disorders. In addition, the validity can be increased by training the model on different and larger datasets. CWT, DCNN and image concatenation can be used in other medical disorders.

## 6. Conclusions

Epilepsy is a common neurological disorder that significantly affects the individual and his/her social environment. The inadequate number of neurological specialists and the global inequality in access to healthcare services emphasise the need for epilepsy diagnosis. Current clinical diagnostic procedures and artificial intelligence methods have limitations. Therefore, there is a need to develop automated systems created with deep learning models for the monitoring and diagnosis of epilepsy. For these reasons, in this study, a new deep learning model and image concatenation method were used to diagnose epilepsy from 35-channel EEG signals. In the publically access dataset used for the study, 35 channels of 1 s each EEG signals were converted into images with CWT. Afterwards, the images belonging to the 35 channels were concatenated and a single image belonging to each sample was obtained. The DCNN model was developed to classify the images. In the last stage, training was performed from the concatenated images without adding any layers to the model and without any different data processing. For the test process, the data that did not participate in the training process were used and a successful classification was made. The developed model and the techniques used can help neurological doctors in determining the most appropriate treatment for epilepsy patients and improving the treatment process. In addition, the quality of life of epilepsy patients can be improved by integrating wearable technologies and health systems.

## Figures and Tables

**Table 1:** Proposed model architecture.

No	Layer Type	Kernel	Activation	Output Shape
1	Rescaling			(150, 210, 3)
2	Conv2D	3 × 3	relu	(148, 208, 4)
3	DepthwiseConv2D	5 × 5	relu	(144, 204, 4)
4	Conv2D	3 × 3	relu	(142, 202, 8)
5	MaxPooling2D	2 × 2		(71, 101, 8)
6	DepthwiseConv2D	5 × 5	relu	(67, 97, 8)
7	MaxPooling2D	2 × 2		(33, 48, 8)
8	Flatten			(12,672)
9	Dropout			(12,672)
10	Dense		relu	(512)
11	Dense		sigmoid	(1)

**Table 2:** Results of the proposed CWT-based DCNN model with different parameters (%).

Input Size	Optimizer	Learning Rate	Accuracy	Sensitivity	Specificity	Precision	F1-Score
50 × 70	Adam	0.0002	81.41	69.51	90.07	83.99	76.06
100 × 140	Adam	0.0002	95.68	94.43	96.62	95.46	94.94
100 × 140	Adam	0.0005	95.60	95.51	95.67	94.51	95.01
150 × 210	Adam	0.0002	95.99	94.28	97.29	96.34	95.30
150 × 210	Adam	0.0001	95.91	94.55	96.98	96.06	95.30
150 × 210	RMSprop	0.0002	93.94	91.90	95.53	94.14	93.01
200 × 280	Adam	0.0002	94.75	92.17	96.73	95.57	93.84

**Table 3:** Comparison of the proposed method with the existing methods (%).

Model	Accuracy	Sensitivity	Specificity	Precision	F1-Score
Transformer [18]	85	87	82	87	87
Hypercube Pattern [12]	87.78	81.32	92.68	89.39	85.16
ResNet50 [53]	66.71	86.40	51.64	59.20	70.25
Xception [51]	87.42	85.65	88.81	85.65	85.65
VGG16 [54]	91.13	89.88	92.10	89.88	89.88
MobileNet [55]	91.66	88.64	94.13	92.46	90.51
InceptionV3 [52]	93.75	89.11	96.99	95.38	92.14
Proposed CWT-based DCNN	95.99	94.28	97.29	96.34	95.30

## Data Availability

https://www.kaggle.com/datasets/buraktaci/turkish-epilepsy (accessed on 20 January 2024).
